# Reversal of spatial memory impairment by phosphodiesterase 3 inhibitor cilostazol is associated with reduced neuroinflammation and increased cerebral glucose uptake in aged male mice

**DOI:** 10.3389/fphar.2022.1031637

**Published:** 2022-12-21

**Authors:** Shuichi Yanai, Tetsuro Tago, Jun Toyohara, Tomoko Arasaki, Shogo Endo

**Affiliations:** ^1^ Aging Neuroscience Research Team, Tokyo Metropolitan Institute of Gerontology, Tokyo, Japan; ^2^ Research Team for Neuroimaging, Tokyo Metropolitan Institute of Gerontology, Tokyo, Japan

**Keywords:** phosphodiesterase 3 inhibitor, cilostazol, memory, neuroinflammation, [^18^F]FDG PET, aging

## Abstract

The nucleotide second messenger 3′, 5′-cyclic adenosine monophosphate (cAMP) and 3′, 5′-cyclic guanosine monophosphate (cGMP) mediate fundamental functions of the brain, including learning and memory. Phosphodiesterase 3 (PDE3) can hydrolyze both cAMP and cGMP and appears to be involved in the regulation of their contents in cells. We previously demonstrated that long-term administration of cilostazol, a PDE3 inhibitor, maintained good memory performance in aging mice. Here, we report on studies aimed at determining whether cilostazol also reverses already-impaired memory in aged male mice. One month of oral 1.5% cilostazol administration in 22-month-old mice reversed age-related declines in hippocampus-dependent memory tasks, including the object recognition and the Morris water maze. Furthermore, cilostazol reduced neuroinflammation, as evidenced by immunohistochemical staining, and increased glucose uptake in the brain, as evidence by positron emission tomography (PET) with 2-deoxy-2-[^18^F]fluoro-d-glucose ([^18^F]FDG). These results suggest that already-expressed memory impairment in aged male mice that depend on cyclic nucleotide signaling can be reversed by inhibition of PDE3. The reversal of age-related memory impairments may occur in the central nervous system, either through cilostazol-enhanced recall or strengthening of weak memories that otherwise may be resistant to recall.

## 1 Introduction

A growing awareness of dementia and age-related cognitive and memory impairment highlights their impact on persons, families, and society, especially aging populations (Prince et al., 2015; Gauthier et al., 2021). Great efforts have been made to better understand the pathological mechanisms of these declines (de Magalhães et al., 2012; Yanai and Endo, 2015; Prickaerts et al., 2017), in particular, the malfunction of the intracellular signaling and transduction that underlies learning and memory (von Linstow Roloff and Platt, 1999).

A variety of signal transduction systems are involved in normal memory formation (Izquierdo and Medina, 1997; Yanai and Endo, 2015). Among them is the 3′, 5′-cyclic adenosine monophosphate (cAMP) pathway, which is long established to play an essential role in memory (for review, see Montminy, 1997; Kandel, 2012; Kida and Serita, 2014). The cAMP-protein kinase A (PKA)-cAMP response element-binding protein (CREB) signaling pathway mediates long-term neuronal plasticity and memory in various species from *Aplysia* (Goelet et al., 1986; Bartsch et al., 1995) to mammals (Florian et al., 2006; Brightwell et al., 2007; Ota et al., 2008). Because the physiological functions served by this pathway decrease in an age-dependent manner (Zimmerman and Berg, 1975; Sugawa and May 1993; Karege et al., 2001), cAMP is likely an ideal molecule to target for the treatment of age-related memory impairment. Recently, strategies centering on the maintenance of pre-senescence cAMP levels have received much attention, with the goal of increasing the intracellular levels of cAMP in a spatiotemporally regulated manner upon memory formation (Heckman et al., 2015; Yanai and Endo, 2019; Sanders and Rajagopal, 2020).

Phosphodiesterases (PDEs) are the only enzymes that degrade cyclic nucleotides including cAMP and/or 3′, 5′-cyclic guanosine monophosphate (cGMP) by breaking their phosphodiester bond (Burgers et al., 1979; Goldberg et al., 1980). The inhibition of PDE leads to elevated level of intracellular cAMP and cGMP concentrations (Omori and Kotera, 2007). The cAMP signaling pathway is suggested to be involved in synaptic plasticity in addition to nitric oxide-cGMP intracellular signaling pathway (Lu and Hawkins, 2002). Further, both cyclic nucleotides are involved in formation of memory (for cAMP, Montminy, 1997; Kandel, 2012; Kida and Serita, 2014; and for cGMP, Fedele and Ricciarelli, 2021; Hollas et al., 2019). Therefore, PDE3 inhibitors, which inhibit the degradation of cAMP and cGMP, are potential candidates for cognitive enhancement medicine. Recent studies have demonstrated that several PDE inhibitors of the PDE superfamily improve or enhance memory and cognitive functions in rodent models (for review, see Reneerkens et al., 2009; Xu et al., 2011; Knott et al., 2017; Yanai and Endo, 2019). One such inhibitor is cilostazol (6-[-4-(1-cyclohexyl-1H-tetrazol-5-yl)butoxy]-3,4-dihydro-2-(1H)-quinolinone), a selective inhibitor of type 3 phosphodiesterase (PDE3) (Yanai et al., 2014; Yanai et al., 2017; Yanai et al., 2018).

Cilostazol is currently prescribed as an antiplatelet agent for the treatment of chronic peripheral arterial occlusion (O'Donnell et al., 2009) and intermittent claudication (Dawson et al., 1998; Chapman and Goa, 2003). The strategy to identify new uses of existing drugs beyond the purpose of their original medical indication is known as drug repositioning (also called drug repurposing or drug reprofiling) (Ashburn and Thor, 2004; Pushpakom et al., 2019). Despite the low level of mRNA expression of PDE3 in human (Lakics et al., 2010) and rodents (Kelly et al., 2014), PDE3 protein is widely distributed in the central nervous system (CNS) (Mitome-Mishima et al., 2013; Maki et al., 2014). Cilostazol also can potentially modulate CNS functions by enhancing the cAMP pathway (Yanai and Endo, 2019). Our previous findings are consistent with cAMP modulation in that long-term cilostazol administration maintains cognitive functions in aging mice (Yanai et al., 2018).

In the present study, we examined whether cilostazol administration reverses already-established memory impairment in aged C57BL/6J mice. For this purpose, we administered cilostazol to 22-month-old C57BL/6J mice that exhibited extensive cognitive impairment (Wolff et al., 2003; von Bohlen und Halbach et al., 2006; Yanai and Endo, 2021), and then we assessed their cognitive function 1 month later when the mice were 23 months old. Further, to determine how cilostazol administration influence brain physiology, we examined cilostazol-induced neuroinflammation and glucose metabolism in the brain using positron emission tomography (PET) with 2-deoxy-2-[^18^F]fluoro-d-glucose ([^18^F]FDG).

## 2 Materials and methods

### 2.1 Ethics statement

All experiments were reviewed and approved by the Animal Experiment Committee of the Tokyo Metropolitan Institute of Gerontology and carried out according to its guidelines (Animal Protocol Approval no. 17012), and also in accordance with *Guide for the Care and Use of Laboratory Animals* (National Research Council (US), 2011). Here, we took special care to report the details of the animal experiments using the Animals in Research: Reporting *In Vivo* Experiments (ARRIVE) guidelines (Kilkenny et al., 2010; Percie du Sert et al., 2020).

The health of mice was monitored daily by animal technicians. In the rare cases when a mouse rapidly lost weight or became emaciated, it was killed humanely. We carefully considered the 3Rs (Russell and Burch, 1959; see also sections below) in designing and carrying out all experiments.

### 2.2 Subjects

Five-weeks-old experimentally naive male C57BL/6J mice were obtained from CLEA Japan Inc (Tokyo, Japan). In the present study, we defined 3-month-old mice as young mice and 23-month-old mice as aged mice. (Yanai et al., 2018). Mice were housed in groups of four to five per cage (Tecniplast S.p.A., Buguggiate (VA), Italy); the cage floors had paper chip bedding. The mice were housed in a specific pathogen free (SPF) vivarium, which was maintained at 22 ± 1°C and 55 ± 5% humidity under a 12-h light-dark cycle (light on at 7:00 a.m.). Periodic examination for the microbial state of the vivarium guaranteed that it was maintained at SPF quality throughout the entire experiment. Mice had free access to standard food (CRF-1; Oriental Yeast Ltd., Tokyo, Japan) and water throughout the entire experiment. The number of mice used in this study and their assignments to the different experimental conditions are presented in Table 1. The experiments were conducted in a blind manner so that the experimenter did not know which mice were administered cilostazol.

**TABLE 1:** Number of subjects used in this study.

Tasks or assay	Young mice	Aged mice (cilostazol %)		
		0% (Control)	0.3%	1.5%
Pharmacokinetic assay^a^ ^,^ ^b^	—	3	14	12
Home-cage activity group^a^	31	8	10	10
Behavioral test battery group^a^				
-Open field	13	16	15	15
-Object recognition	13	16	15	15
-Morris water maze	13	15	15	14
-Pavlovian fear conditioning	13	15	14	14
-Analgesia tests	6	8	7	7
-Immunohistochemical analysis	6	7	7	6
[^18^F]FDG PET^b^	—	7	—	10

^a^ Values are number of subjects in each task or assay in a between subjects design.

^b^ Young mice used as a reference were not given pharmacokinetic assay or [^18^F]FDG PET, assay; and aged subjects receiving 0.3% cilostazol did not receive [^18^F]FDG PET, assay (see Section 2.8).

### 2.3 Apparatus

All apparatuses used in this study were obtained from O’Hara & Co., Ltd (Tokyo, Japan), unless specified otherwise. The open field test and the fear conditioning apparatus were installed in the sound proof rooms. We used the company’s video analysis and automation software Time^®^ (O’Hara &Co., Ltd.) to monitor the mice during behavioral testing in the open field test, Morris water maze, and fear conditioning. Time^®^ was also used to monitor and analyze the home-cage activity of the mice online. The Time^®^ data acquisition system was used to control experimental devices and to analyze behavioral data.

### 2.4 Drug and drug administration

Cilostazol (0%, 0.3%, or 1.5% [w/w]; Shanghai Sunway Pharmaceutical, Shanghai, China; 99.9% purity in HPLC analyses) was mixed in the feed (CRF-1; Oriental Yeast Ltd., Tokyo, Japan). After mice were kept with standard food in the vivarium for 20 months and 3 weeks, i.e., when they reached 22 months of age, they were assigned to one of three dose groups (0, 0.3, or 1.5% cilostazol). We assigned mice in such a way that the mean body weight of each group was comparable. Mice were fed the food-cilostazol mixture *ad libitum* until the end of the experiments. The feeding program for the control mice was the same, except the feed (CRF-1) contained no cilostazol (0% cilostazol). The control (0%) mice served as a non-drug control.

### 2.5 Pharmacokinetic assay

The pharmacokinetics assay for blood concentration of cilostazol was carried out in the 23-month-old mice. Blood was collected at 10 a.m., 2 p.m., or 6 p.m., ensuring that blood cilostazol levels were measured during the course of the daily behavioral experiments, because serum cilostazol concentration varies diurnally in mice receiving mixed-in-feed cilostazol (Yanai et al., 2017). Blood was collected from the caudal vena cava after mice were deeply anesthetized with gaseous isoflurane, and unresponsiveness to all stimuli was confirmed. Blood samples were allowed to clot for 30 min at room temperature before centrifugation for 15 min at 3000×*g* to collect serum. Serum cilostazol concentration was measured using a high-performance liquid chromatography system (Waters Corporation, Milford, MA, United States), according to a method reported previously (Akiyama et al., 1985). Sera from the control (0% cilostazol) mice served as the no-drug baseline.

### 2.6 Behavioral experiments

When mice reached 23 months of age, they were handled approximately 5 min for 3 days by the experimenter, and then they were allocated to one of two groups: home-cage activity group (see Section 2.6.1) or behavioral test battery group (see Section 2.6.2). Mean body weight at the beginning of the behavioral experiments was 42.7 ± 1.1, 45.2 ± 1.0, and 42.1 ± 1.3 g for the control (0%), 0.3%, and 1.5% cilostazol-administered group, respectively. All behavioral experiments were conducted between 10 a.m. and 6 p.m., during the light phase of a 12-h light-dark cycle (light on at 7:00 a.m.). Baseline illumination levels for the open field test and the Pavlovian fear conditioning task were zero, because these apparatuses were installed in the sound proof rooms. For the other behavioral experiments, illumination levels were adjusted at 100 lux (1 m above the floor). Ventilating and air conditioning noise were constantly presented in all behavioral experiment room.

#### 2.6.1 Home-cage activity group

Spontaneous activity (i.e., distance traveled), food, and water intake were measured in the home cages of the mice. Mice were individually housed in cages containing wood-chip bedding (CL-4161; CLEA Japan Inc., Tokyo, Japan). After mice habituated to their new cage for 5 days, we recorded and calculated their spontaneous activity, and food and water intake on the sixth day (Yanai et al., 2018; Yanai and Endo, 2021). Food and water consumption was continuously recorded for each mouse, and the total amount of food and water consumed for the diurnal and the nocturnal phases were obtained on the sixth day.

#### 2.6.2 Behavioral test battery group

Mice were sequentially tested in the open field test, the object recognition task, Morris water maze task, and the Pavlovian fear conditioning task (Table 1). After the completion of fear conditioning, half the mice from each group were tested in a hotplate test, followed by an electrical footshock sensitivity test (Yanai et al., 2018; Yanai and Endo, 2021). For the electrical footshock sensitivity test, a paw flick and vocalizations were used as sensitivity measures. These two tests are referred to as the analgesia tests. The other half of the mice were euthanized by isoflurane and brain tissue was processed for immunohistochemical detection of neuroinflammation (see Section 2.7).

##### 2.6.2.1 Open field test

To examine the locomotor activity and anxiety-like behavior in a novel environment, mice were tested in the open field test as we described previously (Kojima et al., 2008; Yanai et al., 2018; Yanai and Endo, 2021). Briefly, a mouse was placed in the middle of the apparatus (50 × 50 × 30 cm, gray acrylic floor and transparent walls) and allowed to explore freely for 15 min. Behaviors were assessed under a dark condition (10 lx) on the first day and under a bright condition (300 lx) on the second day. Time spent in the center of the apparatus, immobility time, distance traveled (i.e., locomotion), and number of rearings in the apparatus were measured for each mouse.

##### 2.6.2.2 Object recognition task

The object recognition task is a well-established task to study responses of a rodent to spatial changes in the arrangement of objects and to novel objects (Save et al., 1992; Shukitt-Hale et al., 2001). The task was carried out according to procedures detailed in previous studies (Yanai and Endo, 2016b; Yanai et al., 2018). A schematic diagram for the object recognition task is shown in Figure 4A. Mice were individually subjected to six successive, 5 min trials (Trials 1–6). Each trial was separated by a 3-min intertrial interval. On Trial 1, the mouse was allowed to explore the empty arena (50 × 50 cm square arena with a transparent wall, 50 cm in height). In the familiarization trials (Trials 2–4), three salient objects with distinct visual and tactile properties were introduced into the arena before the mouse entered, after which the mouse was allowed to freely explore and interact with the objects. Examples of the objects used were described previously (Yanai and Endo, 2016a). The location of objects remained unchanged throughout the familiarization trials. On Trial 5, the mouse was tested in an object location test, in which one of the familiar objects was moved to a previously unoccupied position in the arena. On Trial 6, a novel object test was performed. In this test, one of the familiar objects was removed and replaced with a new object that was never before introduced into the task and likely never seen before the mouse. This novel object was positioned at the same location as the familiar one it replaced. In each test, the mouse’s exploration of the object was assessed individually by counting the number of physical contacts the mouse made with the object. One contact was counted for each time the mouse’s snout or forepaw touched the object.

##### 2.6.2.3 Morris water maze task

Spatial memory was tested in the Morris water maze task (Morris, 1981; Morris et al., 1982). We tested mice as described previously (Yanai et al., 2018; Yanai and Endo, 2021). During acquisition training, mice were allowed to swim freely for a maximum of 60 s in the pool (100 cm in diameter) in an effort to find the submerged platform. Four acquisition trials were conducted each day for 12 consecutive days. The intertrial interval was 15 min. One day after the completion of acquisition training, a 60-s probe test was conducted with the platform removed from the pool. This probe test assessed memory for the platform location that was learned during acquisition training. During the probe test, the number of swimming traverses (crossings) over the platform’s training location and the time spent in the pool quadrant where the training platform was located were indices of spatial memory (Morris, 1981; Morris et al., 1982). In the cued task following the probe test, the location of the submerged platform was prominently marked by attaching a black flag to it.

##### 2.6.2.4 Pavlovian fear conditioning task

Mice were tested to examine conditioned fear to both a specific cue and testing context, as described previously (Kojima et al., 2008; Borlikova and Endo, 2009; Yanai et al., 2018; Yanai and Endo, 2021). Briefly, mice were individually placed in a shocking chamber on the first day. After an exploratory period of 60 s, the conditioned stimulus (CS; 10 kHz, 70 dB pure tone) was presented for 3 s. The CS co-terminated with an unconditioned stimulus (US; 0.12 mA scrambled electrical footshock for 0.5 s) (conditioning). After the conditioning, mice were consecutively tested for short-term and long-term cue-dependent fear memory. The delay between the conditioning and the first test was 1 h (short-term memory), and the delay for the second test was 24 h (long-term memory). Despite the age-related hearing loss reported in C57BL/6J mice (Yu et al., 2011), aged mice show significant conditioned fear in our protocol (Yanai et al., 2018; Yanai & Endo, 2021). Finally, long-term contextual fear memory was tested at a delay of 48 h.

For the cue-dependent fear memory tests, mice were placed individually into a new chamber to which they were previously unexposed. The chamber used for the cue-dependent memory test was different from the shocking chamber in perspective of brightness, texture, and tactile. In addition, odor stimulus was used in the cue-dependent fear memory test for additional experimental context discrimination. This procedure was used to separate out the contributions of cue-dependent fear memory from context-dependent fear memory; the tone was presented for 60 s. Preliminary experiments confirmed that the experience at short-term cue-dependent memory test (1 h) have no effect on long-term cue-dependent memory test (24 h). For the context-dependent fear memory test, mice were placed in the original shocking chamber in which they received the shocks, but for this test, they received no footshock. Throughout the experiments, freezing duration was measured and used as an index of fear (Fanselow, 1990; Phillips and LeDoux, 1992). We used the established condition that provides the conditioned freezing for cue/context sequence and context/cue sequence (Yanai et al., 2014; Yanai et al., 2018; Kishimoto et al., 2019; Takahashi et al., 2019; Yanai and Endo, 2021).

##### 2.6.2.5 Analgesia tests

After the completion of the fear conditioning task, half of the mice (Table 1) from each group were tested in analgesia tests (hotplate test and the electrical footshock sensitivity test), as described previously (Yanai et al., 2018; Yanai and Endo, 2021).

### 2.7 Immunohistochemical analysis

We measured tissue density of microglia and astrocytes, both of which become activated in neuroinflammation (Glass et al., 2010). After the completion of the context-dependent fear memory test (see also Section 2.6.2.4), mice were returned to their home cage. Thirty minutes later, 6 to 7 mice from each group (Table 1) were prepared for immunohistochemical studies. The mice were deeply anesthetized with isoflurane and transcardially perfused with PBS, 0.01 M phosphate buffer (pH 7.4) containing 0.15 M NaCl, followed by 4% paraformaldehyde in PBS. The brains were removed from the skull and cryoprotected in 30% sucrose in PBS.

Brains were coronally sectioned at 30 µm thickness using a cryostat (Leica Biosystems, Wetzlar, Germany). Sections were washed and subjected to blocking of endogenous peroxidase with 0.3% H_2_O_2_ and 0.3% normal goat serum (NGS; Vector Laboratories, Burlingame, CA, United States) in PBS for 10 min, followed by blocking of non-specific binding and permeabilization with 1.5% NGS and 0.3% Triton in PBS for 1 h. Blocked sections were then incubated overnight at 4°C with anti-ionized calcium-binding adapter molecule 1 (Iba1) antibody (Fujifilm Wako Pure Chemical Corporation, Osaka, Japan), a marker for microglia, or anti-glial fibrillary acidic protein (GFAP) antibody (Abcam, Fremont, CA, United States), a marker for astrocyte diluted in PBS containing 0.5% NGS and 0.3% Triton. Immunoreactions were visualized using an ABC kit (Vector Laboratories) and 3,3′-diaminobenzidine as a substrate.

To quantify Iba1-and GFAP-positive cells in each section, digital images were captured by using the virtual whole-slide imaging system (NanoZoomer 2.0-RS; Hamamatsu Photonics, Hamamatsu, Japan). The numbers of Iba1-and GFAP-positive cells in CA1, CA3, and dentate gyrus (DG) of the hippocampal formation and cerebral cortex were quantified separately with ImageJ Fiji (Schindelin et al., 2012). Nissl-stained adjacent sections were used to identify the region of interest (Figure 8A). For cerebral cortical count, two areas (400 × 400 μm) were randomly selected from each hemisphere of the section (Figure 8B; Zhang et al., 2017). The numbers of Iba1-and GFAP-positive cells were normalized to the cross-sectional area of the region of interest and expressed as counts per mm^2^. Three to four independent sections per mouse were analyzed, and the average counts were used as a representative value of either microglia and astrocytes.

### 2.8 [^18^F]FDG PET group

To examine the effect of cilostazol on neuronal activity in the aged CNS, we prepared separate groups of aged mice. Because oral administration of 0.3% cilostazol had no effect on performance of the behavioral tasks (see Figures 4B, 5C), we compared the uptake of [^18^F]FDG in only aged mice that received 0% or 1.5% cilostazol, as described previously (Yanai et al., 2017). Briefly, before the blood glucose measurement (Stat strip XP2, Nipro, Osaka, Japan), mice were deprived of food overnight. Then, they were intraperitoneally injected with 35.8 ± 1.8 MBq of [^18^F]FDG dissolved in saline. The mice were in the prone position under 1.5%–2.0% isoflurane anesthesia during the entire scanning period. Forty minutes after [^18^F]FDG injection, PET scans of the brain were acquired for 12 min using a semiconductor small animal PET scanner (MIP-100; Sumitomo Heavy Industries, Tokyo, Japan). The PET image data were normalized with a [^18^F]FDG PET template (Mirrione et al., 2007) using standard software (PMOD, version 3.409; PMOD Technologies, Zurich, Switzerland).

[^18^F]FDG uptake in each brain region was corrected for the injected [^18^F]FDG dose and body weight and expressed as standardized uptake value (SUV; activity measured per mL of tissue/injected activity/Gram body weight). T1-weighted magnetic resonance images (MRI) of a representative mouse were also obtained with an ICON (1T; Bruker, Billerica, MA, United States) to be used for superimposing PET-MRI images.

### 2.9 Statistical analysis

Reduced motor activity that accompany normal aging can be a confounding factor in teasing out declines in certain cognitive behaviors from those related purely to reduced motor activity (Wimmer et al., 2012). In animals, performance in memory tasks depend partly on physical factors such as muscle strength and endurance, factors that typically decline with aging (Doherty, 2003; Yanai & Endo, 2021). In line with previous studies, we also observed apparent differences in the physical ability of young and aged mice. Thus, it is difficult to appropriately compare the cognitive function of young and aged mice by using tasks that depend on the animal’s physical capability (Yanai et al., 2018; Yanai and Endo, 2021). For these reasons, all statistical analyses were performed only on data from the three groups of aged mice. Summary data for the young mice (3 months old) are shown only as a “reference” in the figures. Thus, their data were not included in the statistical analyses (see Yanai et al., 2018).

To estimate the required sample size for each group, *a priori* power analysis was performed using G*Power software (Erdfelder et al., 1996; Faul et al., 2007; http://www.gpower.hhu.de/). We calculated that we would need a total sample size of 66 mice to detect a statistically relevant effect of cilostazol administration with 80% actual power, assuming that we used a one-way ANOVA with a significance level of 0.05. We reduced the sample size of the behavioral test battery group to 46 (Table 1), considering the aims of the 3Rs (Russell and Burch, 1959) in using as few mice as possible. We previously found such a reduced sample size was sufficient to test our hypothesis in a similar study (Yanai et al., 2018).

All data were expressed as means ± S.E.M. Prior to examine the statistical difference between/among groups, *F*-tests were performed for each data set and confirm that the means of a given data set have the same standard deviation. Statistical differences among groups were determined by parametric one-way or two-way ANOVA as indicated. All analyses were performed using IBM SPSS Statistics for Windows, version 22 (IBM Corp., Armonk, NY, United States). Statistical significance was set at *p* < 0.05. When a main effect was statistically significant in the ANOVA analysis, Tukey-Kramer multiple comparison tests were conducted to determine which groups differed. Details of the statistical analyses of all experimental data are provided in the Supplementary materials (Supplementary Table S1).

## 3 Results

### 3.1 Serum cilostazol concentration

**FIGURE 1:**
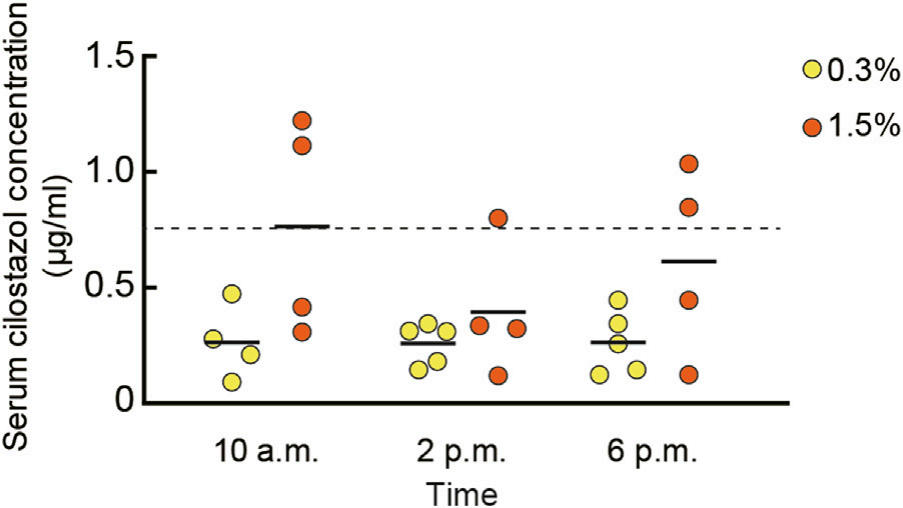
Scatter dot plot representing the pharmacokinetics of cilostazol in blood. Blood serum cilostazol concentration in 23-month-old mice was measured after cilostazol administration for 1 month. Blood was collected at 10 a.m., 2 p.m., or 6 p.m. Two-way ANOVA revealed that mean cilostazol concentration in the 1.5% cilostazol group was significantly higher than that in the 0.3% cilostazol group (*p* < 0.01). Horizontal dashed line at 0.76 μg/ml represents the clinically effective serum concentration in humans (Takase et al., 2007). Although the serum cilostazol concentration in 1.5% cilostazol group appeared lower than clinically effective threshold at 2 p.m. and 6 p.m., this diurnal variation was not statistically significant. Four to five mice were used to determine the serum concentration for each cilostazol concentration and time point. As a control, our assay measured 0 g/ml of serum cilostazol in the group that received 0% oral cilostazol. Horizontal bars indicate mean values.

To determine the concentration of circulating cilostazol, we measured cilostazol concentration in blood serum from mice orally administered 0.3% or 1.5% cilostazol for 1 month (Figure 1). Overall, for the 0.3% cilostazol group, the mean serum cilostazol concentration was similar across all three time points and was consistently below the clinically effective threshold (0.76 μg/ml) in humans that reduces platelet coagulation through the inhibition of PDE3 (Takase et al., 2007). The mean serum cilostazol concentration for the 1.5% cilostazol group was significantly higher than that in the 0.3% cilostazol group (*F* (1, 20) = 8.76, *p* < 0.01). At the 10 a.m. sample time, serum cilostazol concentration reached the clinically effective threshold for mice administered 1.5% cilostazol. Although the serum concentration in this group appeared lower at 2 p.m. than at 10 a.m. and 6 p.m., this diurnal variation was not statistically significant (*F* (2, 20) = 0.95, n. s.).

### 3.2 Spontaneous home-cage activity, food intake, and water intake

**FIGURE 2:**
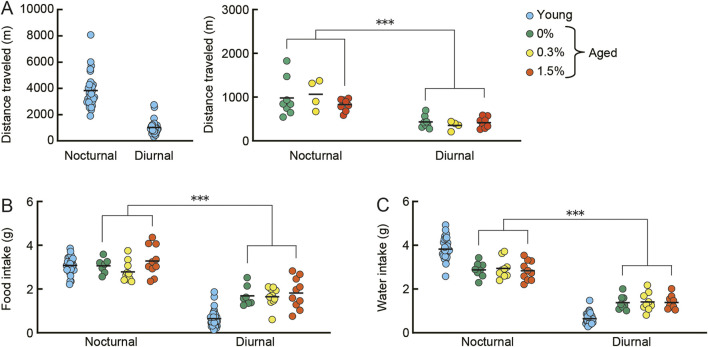
Cilostazol has no effect on spontaneous home-cage activity, food intake, and water intake. Young mice were 3 months old (*n* = 32), and aged mice were 23 months old (*n* = 28). Eight (control) to ten (0.3 and 1.5% cilostazol) aged mice were used for the home-cage observations. Scatter dot plot representing spontaneous activity, i.e., distance traveled **(A)**, food intake **(B)**, and water intake **(C)** on the sixth day for diurnal and nocturnal phases. Due to marked differences in distance traveled between young and three groups of aged mice, data for young and aged mice are shown in separate panels. Two-way ANOVA showed that the aged mice of the three dose groups became active during the nocturnal phase compared to the diurnal phase (distance traveled, *p* < 0.001). We observed the same pattern for food intake (*p* < 0.001) and water intake (*p* < 0.001). Cilostazol administration, however, had no significant effect on any of the indices. Data for young mice are for reference only; they were not included in statistical analysis. ***p < 0.001. Due to equipment failure, activity data for four control (0%) and two aged mice were lost; data for food intake for one control (0%) mouse and data for water intake for one aged mouse (0.3%) were lost. Horizontal bars indicate mean values.

Regardless of the cilostazol concentration administered (0, 0.3, or 1.5%), mice were active in the initially novel home-cage environment. With time, their activity gradually decreased and reached an asymptotic level over 5 days (data not shown). Two-way ANOVA revealed that the aged mice of the three dose groups became significantly active in the nocturnal phase compared to the diurnal phase (Figure 2A; *F* (1, 17) = 89.10, *p* < 0.001). We observed the same pattern for food intake (Figure 2B; *F* (1, 24) = 103.64, *p* < 0.001) and water intake (Figure 2C; *F* (1, 24) = 178.22, *p* < 0.001). Cilostazol administration had no significant effect on any of the indices (activity: *F* (2, 17) = 0.40, n. s.; food intake: *F* (2, 24) = 1.47, n. s.; water intake: *F* (2, 24) = 0.17, n. s.). Not surprisingly, all aged mice appeared to be less active than the young mice, although we did not assess this statistically (see Section 2.9 for the reasoning).

### 3.3 Open field test

**FIGURE 3:**
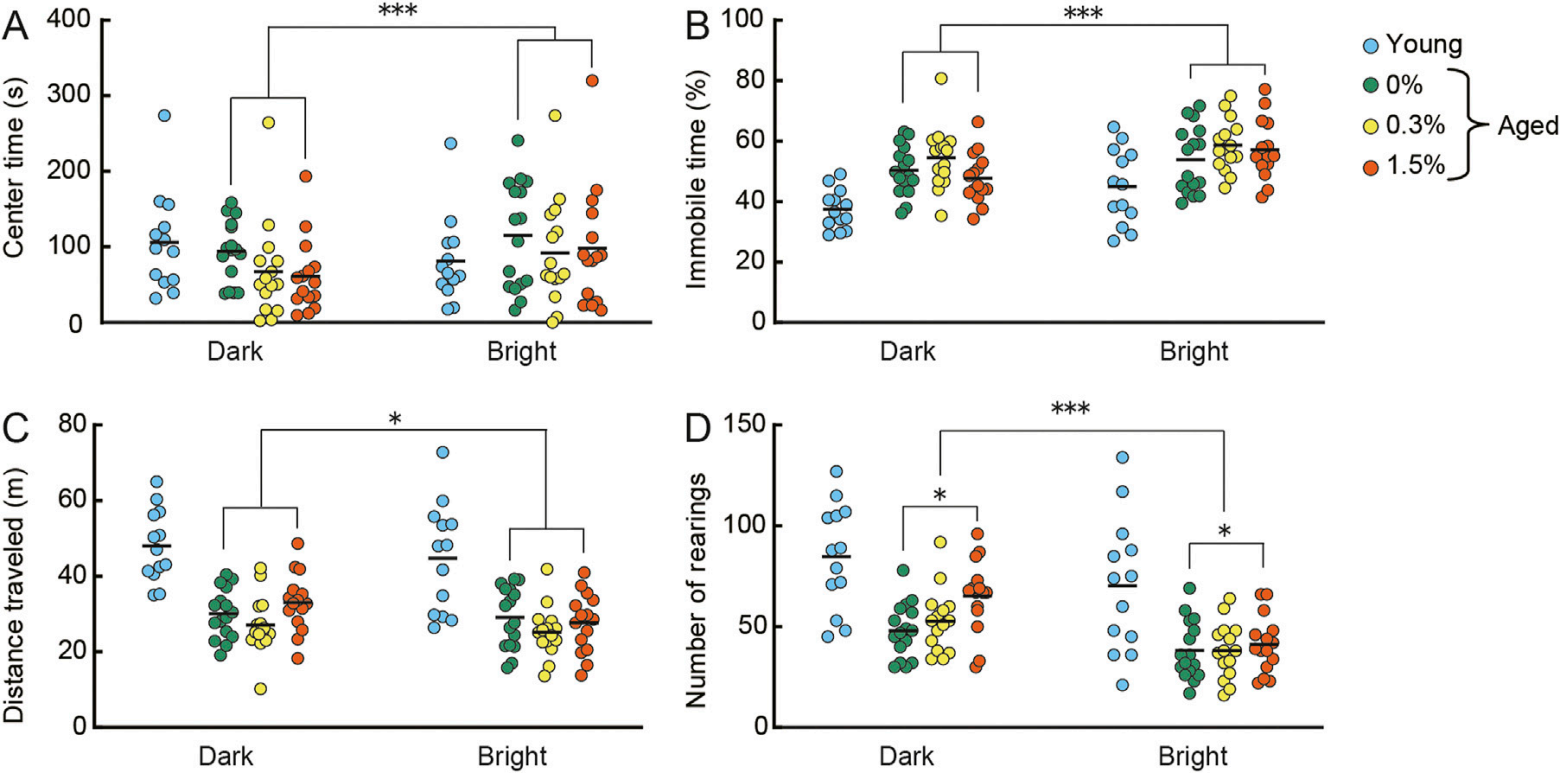
Cilostazol has no effect on anxiety-like behavior, but at higher doses, may enhance locomotor behavior in an open field. Scatter dot plot representing the effect of one month-administration of cilostazol and light condition in the open field test. Anxiety-like behavior [**(A)**, mean time spent in center of the apparatus; **(B)**, mean immobility time] and locomotor activity [**(C)**, mean distance traveled; **(D)**, mean number of rearings] were assessed under dark conditions on the first day and under bright conditions on the second day. Statistical differences among groups were determined by two-way ANOVA. Regardless of cilostazol dose administered, the time spent in the center (*p* < 0.001) and immobility time (*p* < 0.001) were significantly greater on the second day compared to the first day. The results suggest that anxiety state in aged mice is unaffected by 1 month of cilostazol administration and that aged mice in the three dosage groups responded similarly in both dark and bright conditions. For the locomotor activity indices, the three dose groups showed significantly reduced locomotor activity on the second day compared to the first day in perspective of distance traveled (*p* < 0.01) and the number of rearings (*p* < 0.001). We observed a significant main effect of cilostazol dose for the number of rearings (*p* < 0.05), showing that the number of rearings of the 1.5% cilostazol group was significantly greater than that of the control (0%) group. 0%, 0.3%, and 1.5% represent the concentration of orally administered cilostazol. Performance of 3-month-old young mice (Young) is shown as a reference but not included in statistical analyses. ***p < 0.001, *p < 0.05. Horizontal bars indicate mean values.

In the open field test (Figure 3), the amount of time spent in the center of the arena and average immobility time in the arena are measures of a mouse’s anxiety level, whereas distance traveled and the number of rearing episodes are measures of locomotor activity (Walsh and Cummins, 1976). These behaviors were assessed under dark conditions on the first day and under bright conditions on the second day in the open field. In general, aged mice are more anxious and less mobile compared to young mice. Regardless of cilostazol dose administered, the time spent in the center (Figure 3A) and immobility time (Figure 3B) were significantly greater on the second day compared to the first day (center time: *F* (1, 43) = 11.79, *p* < 0.001; immobility time (*F* (1, 43) = 23.83, *p* < 0.001). The effect of light condition on center time is not replicable in previous studies using aged mice (Yanai et al., 2018; Yanai & Endo, 2021). This inconsistency of behavior might be something to do with aging, further examination is required to validate center time in our experimental condition. The results suggest that anxiety state in aged mice is unaffected by 1 month of cilostazol administration and that aged mice in the three dosage groups responded similarly in both dark and bright conditions.

For the locomotor activity indices (Figures 3C, D), the three dose groups (0, 0.3%, and 1.5%) showed significantly reduced locomotor activity on the second day compared to the first day (distance traveled: *F* (1, 43) = 7.60, *p* < 0.01; number of rearings: *F* (1, 43) = 30.73, *p* < 0.001). We observed a significant main effect of cilostazol dose for the number of rearings (*F* (2, 43) = 3.39, *p* < 0.05). Tukey-Kramer multiple comparison tests revealed that the number of rearings of the 1.5% cilostazol group was significantly greater than that of the control (0%) group (*p* < 0.05). Taken together, the results suggest that cilostazol has no major effect on anxiety-like behavior, but at higher doses, it may enhance locomotor behavior. Although traditional interpretations of behavior in the open field test state that distance traveled and the number of rearings made reflect locomotor activity (Walsh and Cummins, 1976), a recent study noted that the number of rearings reflects explorative behavior rather than simple locomotor activity (Lever et al., 2006). Further research using other anxiety-and-mobility tasks, like the hole board exploration task (Bradley et al., 1968; File and Wardill, 1975; Barnes, 1979; van Gaalen and Steckler, 2000) is necessary to clearly reveal the influence of cilostazol on exploratory behavior in aging.

### 3.4 Object recognition task

**FIGURE 4:**
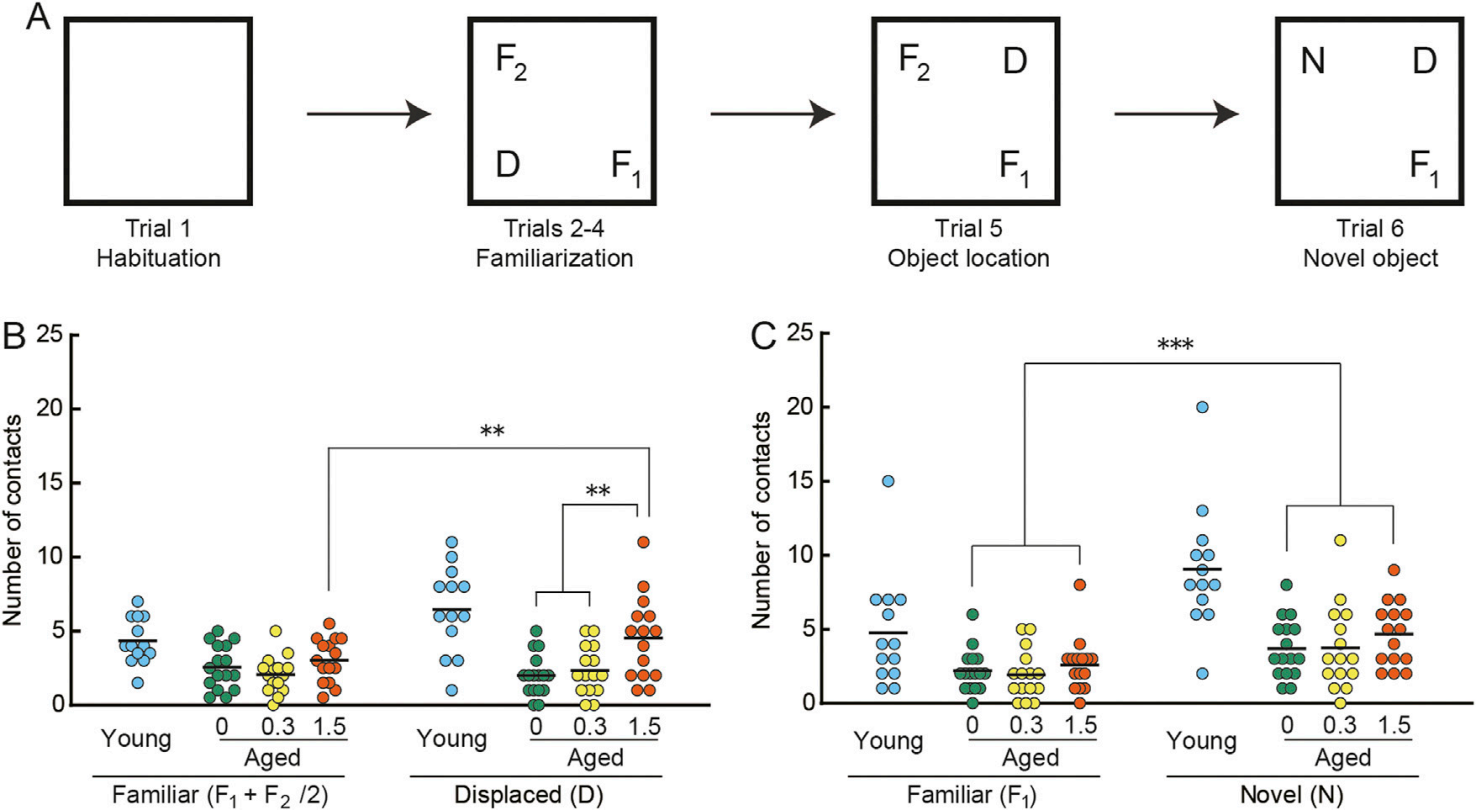
Cilostazol improves novel recognition memory in aged mice. **(A)** Schematic diagram of object recognition task and apparatus. After the mouse habituated in the empty arena (Trial 1) and became familiar with the three objects (F_1_, F_2_, D) placed in the arena (Trials 2–4), it was consecutively tested first on the object location test (Trial 5), in which one of the familiar objects (D) was displaced, and then on the novel object test (Trial 6), in which a familiar object (F_2_) was replaced by a novel (N) one. Statistical differences among groups were determined by two-way ANOVA. **(B)** Scatter dot plot representing number of contacts in the spatial change test (Trial5). Among the three groups of aged mice, only the 1.5% cilostazol group made more contacts with the displaced object than with the familiar objects, showing a significant preference toward the displaced object (*p* < 0.01). **(C)** Scatter dot plot representing number of contacts in the object change test (Trial 6). Mice of the three dose groups intensively explored the novel object (*p* < 0.001). However, there were no significant differences among the three groups. For panels B and C, 0, 0.3, and 1.5 represent the concentration (percentage) of orally administered cilostazol. Performance of 3-month-old young mice (Young) is shown as a reference but not included in statistical analyses. ***p < 0.001, ** *p* < 0.01. Horizontal bars indicate mean values.

Behavioral responses to changes in the spatial arrangement of objects and to a novel object were examined in the object recognition task (Save et al., 1992; Shukitt-Hale et al., 2001; Yanai et al., 2018). We observed apparent differences in the number of object contacts made by young mice compared to those by mice in the three aged mice groups, both in the object location test (Figure 4B) and the novel object test (Figure 4C). These differences may reflect an apparent reduction in basal activity observed in aged mice under novel conditions (Figure 3C) and in their home-cage environment (Figure 2A, see also Section 3.2).

In the object location test (Figure 4B), the control (0%) and 0.3% groups made a similar number of contacts to both displaced and familiar objects. On the other hand, the 1.5% cilostazol group made more contacts with the displaced object than with the familiar objects, showing a preference toward the displaced object. Two-way ANOVA revealed that the main effect of cilostazol dose was significant (*F* (2, 43) = 5.18, *p* < 0.01). We also observed a significant interaction between cilostazol dose and object category (*F* (2, 43) = 5.48, *p* < 0.01). A test for the simple main effect showed a significant preference toward the displaced object in the 1.5% group (*p* < 0.01), whereas the other two groups of aged mice did not show an object preference. The results show that 1 month of 1.5% cilostazol administration improves novel recognition spatial memory in aged mice. Since this kind of memory task is dependent on the hippocampus and associated brain structures (Warburton et al., 2013; Barker and Warburton, 2015), hippocampal function may have been enhanced by cilostazol.

By contrast, mice of the three dose groups intensively explored the novel object (Figure 4C), as indicated by the significant main effect of object category (*F* (1, 43) = 31.73, *p* < 0.001). However, there were no significant differences among the three groups (*F* (2, 43) = 0.96, n. s.), suggesting that the doses of cilostazol we used had no apparent effect on the visual acuity required to discriminate objects (Yanai et al., 2014; Yanai et al., 2018).

### 3.5 Morris water maze task

**FIGURE 5:**
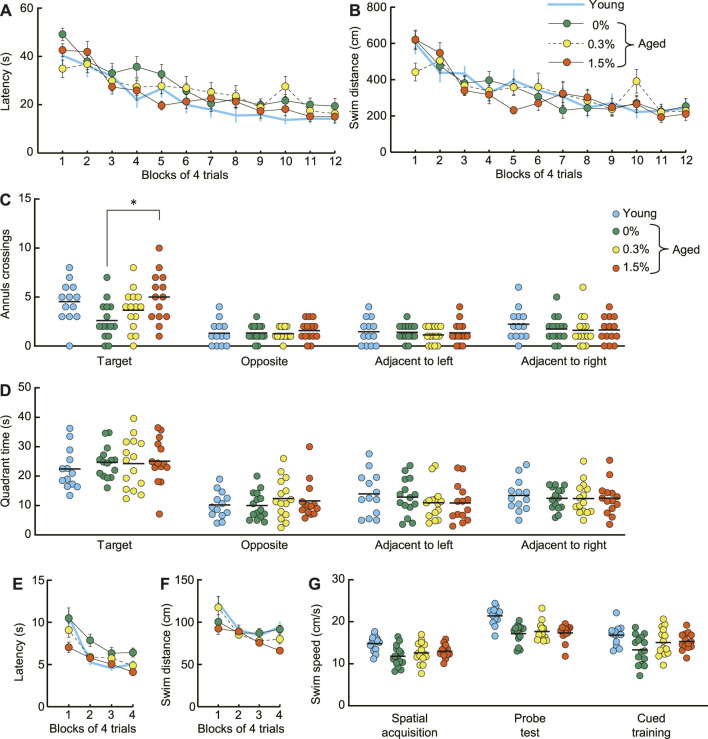
Cilostazol improves spatial memory and cued learning of aged mice in the Morris water maze. **(A)** Mean escape latency and **(B)** mean swim distance to the submerged platform decreased at a similar rate during acquisition training. Two-way ANOVA showed that performance over 12 days of acquisition training was similar for the three dosage groups of aged mice. **(C)** Scatter dot plot representing number of platform crossings (annulus crossings) during the probe test, which was conducted 1 day after the completion of spatial acquisition training. The 1.5% dosage group made significantly greater number of swimming traverses over the previous platform position than that of the control (0%) mice (*p* < 0.05). **(D)**Scatter dot plot of time spent in the training quadrant during the probe test. Mean time spent swimming in the training quadrant during the probe was similar among the different cilostazol dosage groups. **(E)** Mean escape latency and **(F)** mean swim distance to the visible platform during the visual cued training in the Morris water maze. The 1.5% cilostazol group had significantly faster escape latency than control (0% cilostazol) aged mice (*p* < 0.01). The 1.5% dosage group also had shorter swim distances, however, this difference was marginally significant (*p* = 0.103). **(G)** Scatter dot plot representing swim speed during acquisition training, probe test, and cued training. The swimming speed was equivalent among the three dosage groups throughout the task. For **(A–G)**, 0%, 0.3%, and 1.5% represent the concentration of orally administered cilostazol. Performance of 3-month-old young mice (Young) is shown as a reference but not included in statistical analyses. * *p* < 0.05 compared with the control mice. Error bars in **(A,B,E,F)** indicate S.E.M. Horizontal bars in **(C,D,G)** indicate mean values.

During spatial memory learning (acquisition), both the mean escape latency and the swimming distance to reach the submerged platform decreased similarly for mice receiving 0%, 0.3%, or 1.5% cilostazol (Figures 5A, B). The main effect of cilostazol dose was not significant (latency: *F* (2, 41) = 1.09, n.s.; swim distance: *F* (2, 41) = 0.12, n.s.). The results show that the acquisition of spatial memory, which is dependent on an intact hippocampus (Morris et al., 1982), is unaffected by 1 month of cilostazol administration. Also, swimming ability in aged mice appeared to be unaffected by cilostazol, because the average swimming speed was equivalent among the three dosage groups during task training (Figure 5G; acquisition, *F* (2, 41) = 1.10, n. s.; probe test, *F* (2, 41) = 0.21, n.s.; cued training, *F* (2, 41) = 1.95, n.s.).

In the probe test, aged mice receiving no cilostazol showed fewer platform crossings compared to young mice (Figure 5C), confirming previous findings that spatial memory is impaired in 23-month-old C57BL/6J mice compared to young mice (Yanai et al., 2018; Yanai and Endo, 2021). Cilostazol administration significantly improved memory for the training position of the platform during task acquisition, as measured by swimming traverses over its previous position. The mean number of platform crossings in aged mice receiving cilostazol was significantly greater compared to those aged mice receiving none (*F* (2, 41) = 4.40, *p* < 0.05). Post hoc comparison revealed that the number of crossings in the 1.5% dosage group was significantly greater than that of the control (0% cilostazol) aged mice (*p* < 0.05). Importantly, average memory performance of 23-month-old mice in the 1.5% cilostazol group was comparable to that of young mice (Figure 5C). The other *post hoc* comparisons were not statistically different (0.3% cilostazol vs control: *p* = 0.38; 0.3% vs. 1.5% cilostazol: *p* = 0.24). In contrast to platform crossings during the probe trial, mean time spent swimming in the training quadrant during the probe was similar among the different cilostazol dosage groups (Figure 5D; *F* (2, 41) = 0.05, n.s.).

In the cued training, the 1.5% cilostazol group had significantly faster escape latency than control (0% cilostazol) aged mice (Figure 5E; *F* (2, 41) = 5.26, *p* < 0.01). The 1.5% dosage group also had shorter swim distances (Figure 5F). However, this difference was marginally significant (*F* (2, 41) = 2.41, *p* = 0.103). Impaired performance of aged control mice in the cued training on the Morris water maze could be an indication of visual impairment. However, this explanation is unlikely since the aged control mice and the 1.5% and 0.3% dosage groups performed similarly in the novel object test of the object recognition task (Figure 4C). Therefore, superior performance of the cued training we observed in aged mice receiving 1.5% cilostazol may reflect true facilitation in visual cued learning in the Morris water maze, specifically facilitation of learning new rules of the task (Steele and Morris, 1999).

### 3.6 Pavlovian fear conditioning task

**FIGURE 6:**
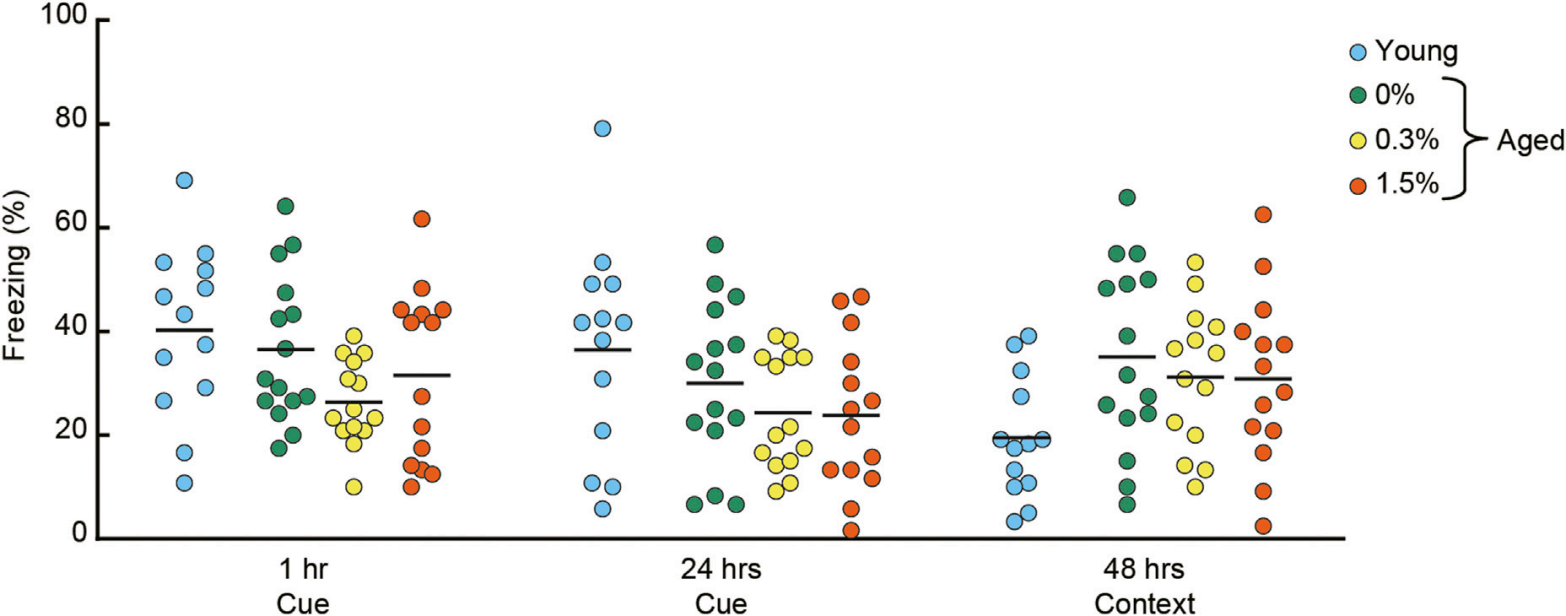
Cilostazol has no effect on Pavlovian fear conditioning. After conditioning with a tone CS and shock US, conditioned freezing was sequentially assessed for cue-dependent fear memory (1 h and 24 h after the conditioning), followed by context-dependent fear memory (48 h after conditioning). Scatter dot plot representing percentage of conditioned freezing. One-way ANOVA showed that aged mice receiving the three cilostazol dosages showed a similar level of freezing throughout the task. In the graph, 0%, 0.3%, and 1.5% represent the concentration of orally administered cilostazol. Performance of 3-month-old young mice (Young) is shown as a reference but not included in statistical analyses. Horizontal bars indicate mean values.

After conditioning with a paired presentation of tone CS and footshock US, mice were sequentially tested for short-term (1 h) and long-term (24 h) cue-dependent fear memory, followed by long-term contextual fear memory (48 h) (Figure 6). Aged mice receiving the three cilostazol dosages showed a similar level of freezing in the short-term cue-dependent fear memory test (*F* (2, 40) = 2.04, n.s.) and long-term cue-dependent fear memory test (*F* (2, 40) = 0.91, n.s.).

In the context-dependent fear memory test conducted 48 h after conditioning, aged mice receiving the three cilostazol dosages (0, 0.3, 1.5%) showed substantially higher levels of freezing compared to young mice. However, regardless of cilostazol dosage, performance among the three groups of aged mice was statistically indistinguishable (*F* (2, 40) = 0.31, n.s.). The basis of the higher overall freezing in the three groups of aged mice may be reduced locomotor activity (Figure 3C) and home-cage activity (Figure 2A). The results show that cilostazol did not affect conditioned fear memory. Recently, we reported that aged mice showed higher freezing compared to young mice (Yanai and Endo, 2021). Because we optimized the fear conditioning protocol for young mice (Yanai et al., 2014), the protocol used in the present study might have led to a ceiling effect, manifested as saturation of freezing behavior in the three groups of aged mice. Sensitivity to aging and/or involvement of hippocampus in the fear conditioning task is often inconsistent among studies (for review, see Kennard & Woodruff-Pak, 2011). The inconsistency may be due to the type (manufacturer) of the shock generator (direct current/alternating current), experimental condition/environment/context. Thus, with this interpretation, any cilostazol effect might have been masked.

### 3.7 Analgesia tests

**FIGURE 7:**
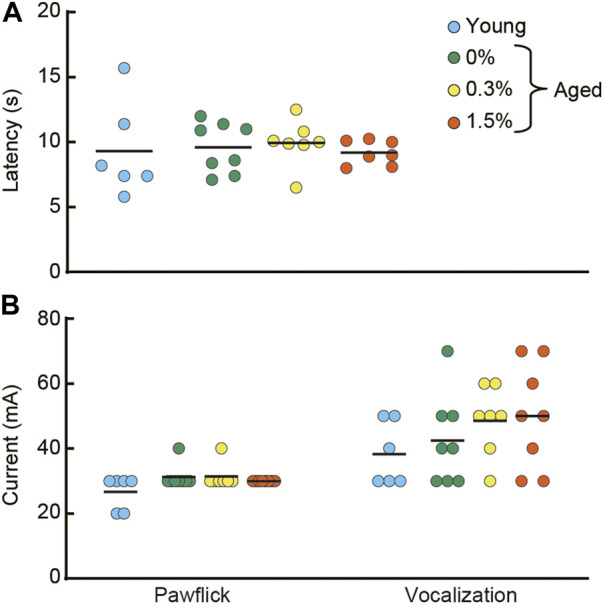
Cilostazol has no effect on pain sensitivity. After the completion of the fear conditioning task, half of the mice from each group were tested in hotplate test and the electrical footshock sensitivity test. **(A)** Scatter dot plot representing latency to lick a forepaw. One-way ANOVA showed that the aged mice receiving 0.3 or 1.5% cilostazol performed similarly to the aged control (0% cilostazol) mice. **(B)** Scatter dot plot of electric current intensity to evoke a pawflick and vocalization. One-way ANOVA revealed no statistical significance among the three groups of aged mice. These results from the two analgesia tests show that 1 month of cilostazol has no apparent effect on pain sensitivity, regardless of the type of noxious stimuli. In the graph, 0%, 0.3%, and 1.5% represent the concentration of orally administered cilostazol. Data for young mice (Young) are for reference only; they were not included in statistical analyses. Horizontal bars indicate mean values.

In the hotplate test and electrical footshock sensitivity test, the aged mice receiving 0.3 or 1.5% cilostazol performed similarly to the aged control (0% cilostazol) mice (Figure 7A; hotplate test: *F* (2, 19) = 0.37, n.s.; Figure 7B; paw flick in electrical footshock sensitivity test: *F* (2, 19) = 0.47, n.s.; vocalization in electrical footshock sensitivity test: *F* (2, 19) = 0.60, n.s.). These results from the two analgesia tests show that 1 month of cilostazol has no apparent effect on pain sensitivity.

### 3.8 Immunohistochemical analysis for neuroinflammation

**FIGURE 8:**
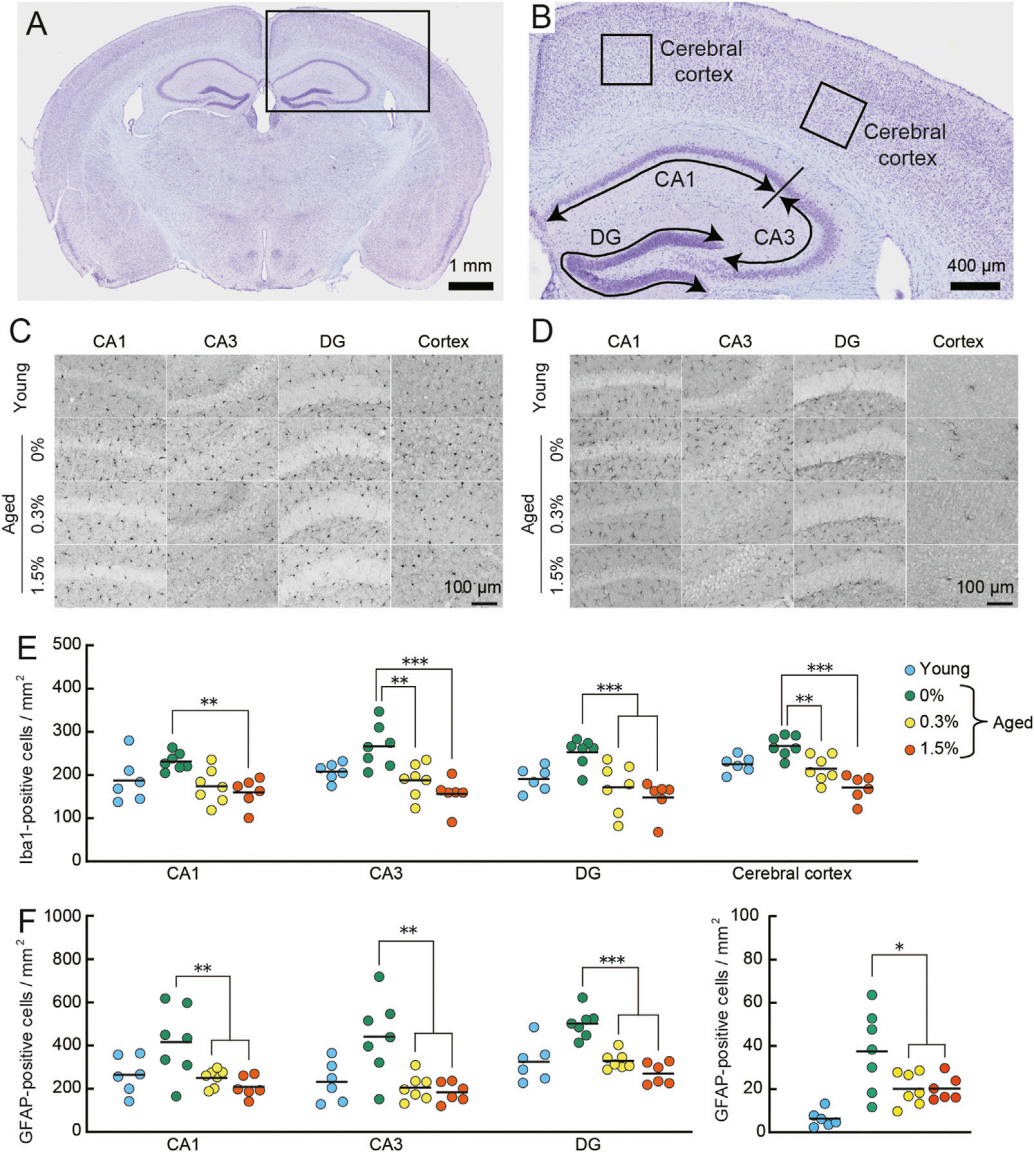
Cilostazol reverses neuroinflammation in aged mice. Mouse brains were processed for immunohistochemical analysis and photo-documentation of Iba1-and GFAP-positive cells in the hippocampus and cerebral cortex after completing the context-dependent fear memory test. **(A)** Light photomicrograph of a Nissl-stained coronal section from an aged mouse showing the analysis area (box) in the brains for quantitation in cerebral cortex and hippocampus. Scale bar is 1 mm. **(B)** Higher magnification photomicrograph of analysis areas in hippocampus (CA1, CA3, DG) and cerebral cortex. Scale bar is 400 μm. **(C,D)** Representative high-magnification images of Iba1-positive **(C)** and GFAP-positive **(D)** cells in the indicated brain regions of an aged mouse receiving the indicated dosage of cilostazol. Scale bars are 100 μm for all images. **(E)** Scatter dot plot representing Iba1-positive cells in subregions of hippocampus and in cerebral cortex. Quantitative analysis using one-way ANOVA showed that 1 month of cilostazol administration significantly reduced the number of Iba1-positive cells in all subregions of the hippocampus (CA1, *p* < 0.01; CA3, *p* < 0.001; DG, *p* < 0.001) and the cerebral cortex (*p* < 0.001). **(F)** Scatter dot plot of GFAP-positive cells. Due to major differences between the cortex and other hippocampal region, data for cerebral cortex is shown in an independent panel. One-way ANOVA showed that the mean number of GFAP-positive cells in aged mice receiving cilostazol was significantly lower than that of the control aged mice (CA1, *p* < 0.01; CA3, *p* < 0.001; DG, *p* < 0.001; cerebral cortex, *p* < 0.05). For **(C–F)**, 0%, 0.3%, and 1.5% represent the concentration of orally administered cilostazol. Data from 3-month-old young mice (Young) are shown as a reference but not included in statistical analyses. ***p < 0.001, **p < 0.01, *p < 0.05 compared with the control aged mice. Horizontal bars in panel E and F indicate mean values.

Several lines of evidence show that cAMP has an inhibitory effect on T-cell proliferation (Kammer, 1988), interleukin (IL) production (Barton et al., 1996), and nuclear factor kappa B (NF-κB) activity (Gerlo et al., 2011). Activation of T-cells, IL, and NF-κB initiate events leading to inflammation. In congruent with these studies, several types of PDE inhibitors inhibit inflammation in CNS (Wang et al., 2012; Zhang et al., 2013; Lee et al., 2019). On the other hand, other studies reported the PDE inhibitors-induced inflammation (Pathak et al., 2017; Pilarzyk et al., 2021), leaving it controversial whether the effect of PDE inhibitor is proinflammatory or anti-inflammatory. To examine the effect of cilostazol on neuroinflammation, we examined histological markers of neuroinflammation in aged control mice and aged mice receiving cilostazol. Thirty minutes after the last test in the fear conditioning task, brains were processed for immunohistochemical detection of Iba1 and GFAP. Iba1 is specifically expressed in activated microglia (Ito et al., 1998), while GFAP is expressed in the intermediate filament protein of mature astrocytes (Eng and Ghirnikar, 1994). During activation, astrocytes express more GFAP (Brahmachari et al., 2006). Similar to aged mice in previous studies (Conde and Streit, 2006; Ownby, 2010), aged control mice (0% cilostazol) had more Iba1-and GFAP- positive cells in the hippocampus and cerebral cortex (Figures 8C, D, respectively) compared to young mice. In both the 0.3% and 1.5% cilostazol groups, the aged mice had fewer Iba1-and GFAP-positive cells compared to the control aged mice, and the mean numbers appeared to be comparable to those in young mice.

Quantitative analysis of neuroinflammation in aged control and the two cilostazol dosage groups of aged mice showed that 1 month of cilostazol administration significantly reduced the number of Iba1-positive cells (Figure 8E) in all subregions of the hippocampus (CA1: *F* (2, 17) = 9.21, *p* < 0.01; CA3: *F* (2, 17) = 11.95, *p* < 0.001; DG: *F* (2, 17) = 9.96, *p* < 0.001) and the cerebral cortex (*F* (2, 17) = 19.19, *p* < 0.001). Post hoc comparisons revealed that the mean number of Iba1-positive cells in mice administered cilostazol was significantly lower than that of the control aged mice. The mean numbers of Iba1-positive cells in the 0.3% and 1.5% dosage groups was statistically indistinguishable. A similar result was obtained for GFAP-positive cells, as the main effect of cilostazol administration was significant (CA1: *F* (2, 17) = 7.67, *p* < 0.01; CA3: *F* (2, 17) = 10.46, *p* < 0.001; DG: *F* (2, 17) = 34.28, *p* < 0.001; cerebral cortex: *F* (2, 17) = 4.43, *p* < 0.05) (Figure 8F). Post hoc tests showed that the mean number of GFAP-positive cells in aged mice receiving cilostazol was significantly lower than that of the control aged mice. The brains were collected 30 minutes after the completion of the context-dependent fear conditioning task. It is reported that stress induces neuroinflammation (For review, see Calcia et al., 2016). The degree of stress induced by the procedure is unclear, however, such a condition is the same for all mice processed for the immunohistochemical examination. The results show that 1 month of cilostazol administration leads to decreased levels of neuroinflammation, as evidenced by the numbers of microglia and astrocytes in the hippocampus and cerebral cortex.

### 3.9 [^18^F]FDG uptake in brain

**FIGURE 9:**
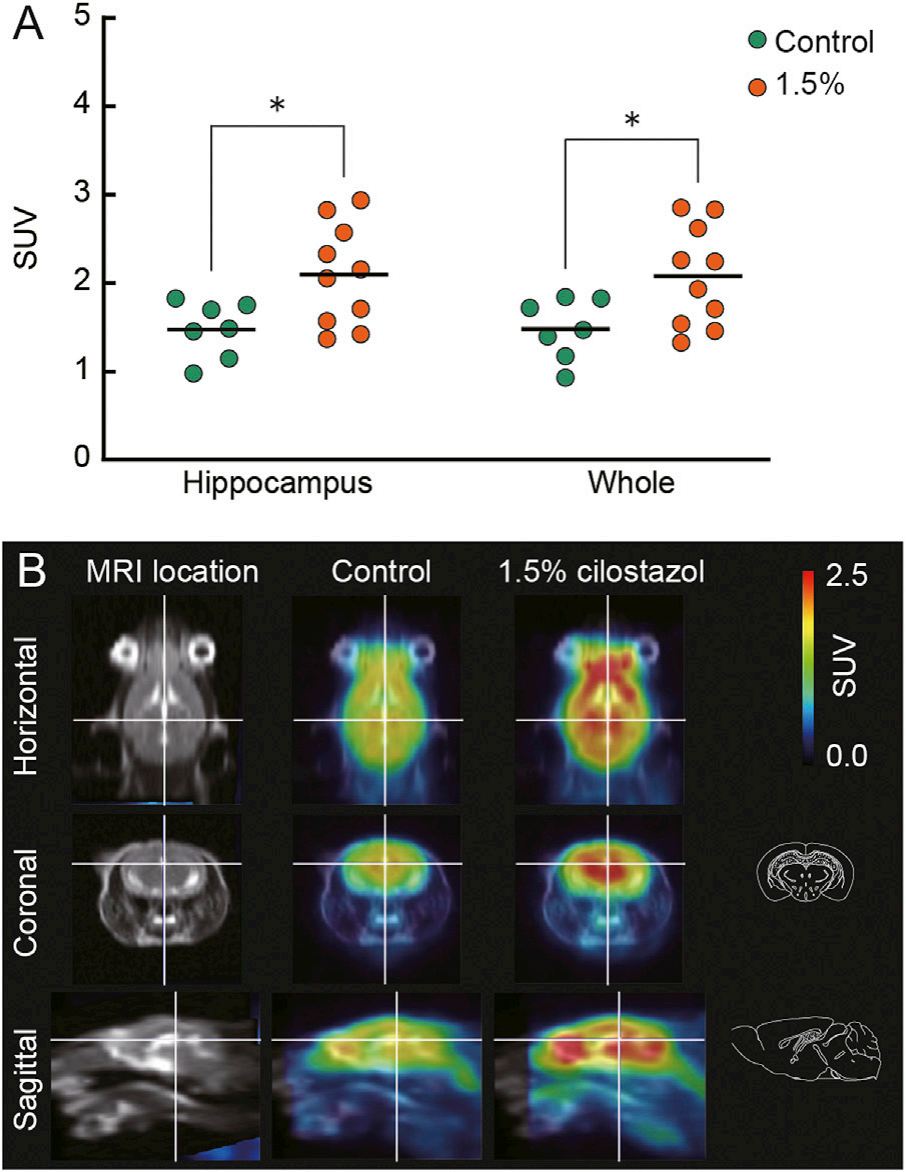
Cilostazol enhances cerebral glucose metabolism in aged mice. Cilostazol was administered to seven control (0%) and nine (1.5% cilostazol) 22-month-old mice for 1 month, and then the uptake of [^18^F]FDG was assessed using PET. **(A)** Scatter dot plot representing the uptake of [^18^F]FDG (SUV) in hippocampus and whole brain (Whole). Student’s t-test revealed that mice administered 1.5% cilostazol showed significantly greater [^18^F]FDG uptake in the hippocampus (*p* < 0.05) and whole brain (*p* < 0.05). *p < 0.05. Horizontal bars indicate mean values.**(B)** Representative T1-weighted magnetic resonance images (MRI) and superimposed PET-MRI image of mouse brains acquired after intravenous injection of [^18^F]FDG (summed frames from 40 min to 52 min). Schematic illustrations of coronal and sagittal sections (Paxinos and Franklin, 2001) corresponding to superimposed PET-MRI images are also shown. For **(A,B)** 0%, or 1.5% represent the concentration of orally administered cilostazol. Blue-to-red colors represent arbitrary unit for lower-to-higher amounts of [^18^F]FDG.

To examine the modulation of neuronal activity by cilostazol, we administered cilostazol (0% or 1.5%) to aged mice for 1 month, and then we assessed the uptake of [^18^F]FDG using PET. Increased [^18^F]FDG uptake reflects neuronal activity in the brain (Bentourkia et al., 2000; Kato et al., 2016). The administration of 1.5% cilostazol significantly increased [^18^F]FDG uptake in the hippocampus (*t* (15) = 2.59, *p* < 0.05) and the whole brain (*t* (15) = 2.46, *p* < 0.05) (Figure 9A) compared with control aged mice who received no cilostazol. See Supplementary Figure S1 for other brain regions analyzed. PET scan data (Figure 9B) for the aged control (0% cilostazol) mice and mice receiving 1.5% cilostazol were consistent with our [^18^F]FDG uptake data (Figure 9A). Several high-uptake areas were observed, a prominent one being the midbrain including hippocampus (Figure 9B). The results suggest that overall cerebral glucose metabolism is enhanced in aged mice receiving 1.5% cilostazol. The competition between endogenous circulating blood glucose and [^18^F]FDG on the glucose transporter resulted in less potential for [^18^F]FDG to uptake (Hamidian Jahromi et al., 2014). Cilostazol administration did not significantly affect blood glucose level for the control group (105.9 ± 10.0 mg/dl) and for the 1.5% cilostazol group (97.4 ± 4.7 mg/dl); *t* (15) = 0.86, n.s.). These results exclude the potential differences of competitive inhibition on [^18^F]FDG uptake by circulating blood glucose (Love et al., 2005).

## 4 Discussion

We previously reported that long-term cilostazol administration, starting from 13 months of age, prevented age-related memory impairment that normally manifests at 23 months of age in male C57BL/6J mice (Yanai et al., 2018). An open question was whether cilostazol could also reverse memory impairment in male C57BL/6J mice that would otherwise be expressed in old age. In the present study, we demonstrated that 1 month of 1.5% cilostazol administration reversed impaired hippocampus-dependent memory in 23-month-old male C57BL/6J mice, the most commonly used animal model of aging research (Mitchell et al., 2015; Yanai and Endo, 2021). Furthermore, we demonstrated that this cognitive amelioration was associated with reduced neuroinflammation and increased uptake of glucose in the brain.

We provided evidence of significantly improved performance of 1.5% cilostazol group in object recognition memory (Figure 4B). In the probe test of the Morris water maze task, three groups of 1.5% cilostazol group had a greater number of platform crossings (Figure 5C) despite equivalent performance in time spent in the training quadrant (Figure 5D). Somewhat surprisingly, reversed memory performance in 23-month-old mice was similar to that of 3-month-old mice young mice. One interpretation of this difference in the two measures is that encoding of the general “neighborhood” location of the platform during training (i.e., quadrant) may be augmented by further spatial information processing that encodes the precise location of the platform within the quadrant (Yanai and Endo, 2021). This difference is sometimes described as a coarse spatial representation *versus* a fine-grained representation about the locations of objects (Evensmoen et al., 2013). The number of platform crossings during a probe trial in the Morris water maze, then, reflects the memory of this more precise fine-grained spatial memory (Yanai and Endo, 2021). With this interpretation, the present results suggest that 1 month of 1.5% cilostazol helped aged mice better encode the exact platform location during training, which was manifested by more precise traverses over the training location of the platform’s previous location during the probe test. Whereas precise spatial memory normally declines with advancing age in human (Nilakantan et al., 2018; Segen et al., 2021), the action of cilostazol may have enhanced the processes responsible for encoding fine-grained spatial memory for exact location (Figures 5C, D). Human aging is associated with a decline in a wide range of cognitive functions (for review, see Salthouse, 2010; Murman, 2015), and one of cognitive function vulnerable to aging is spatial memory (Merhav et al., 2019). Similar to the observation in the present study, age-related decline in human spatial memory is not uniform; human aging is associated with loss of precise spatial memory (McAvan et al., 2021). These results may suggest that the effect of aging on spatial memory processing share a common feature in perspective of its accuracy. Aging is a complex process, and it has been compellingly argued that different organ systems may age at different rates across the whole body (López-Otín et al., 2013; Rando and Wyss-Coray, 2021). Inflammation is linked to many phenomena associated with aging (McGeer and McGeer, 2004; Chung et al., 2009). Furthermore, emerging evidence has revealed the roles of inflammation in different diseases (e.g., Coussens and Werb, 2002; Libby et al., 2002; Hotamisligil, 2006). The cAMP signaling pathway is one pathway that is involved in the suppression of inflammatory responses (Kammer, 1988; Barton et al., 1996; Gerlo et al., 2011). Consistent with other PDE inhibitors (Pearse and Hughes, 2016; Esin et al., 2022), cilostazol reduces the amount of peripheral inflammatory cytokines such as tumor necrosis factor-α (TNF-α) (Li et al., 2017) and members of the IL family (Sakamoto et al., 2018), and the expression of their downstream target, NF-κB (da Motta and de Brito, 2016).

In the present study, we expanded these inflammation-related observations to include similar effects in the CNS (Figures 8E, F). Specifically, cilostazol reduced the numbers of Iba1-and GFAP-positive cells in hippocampus; these reactive cell types are indicative of neuroinflammation (Sankar et al., 2019; Thadathil et al., 2021). Considering that memory impairment have been observed following neuroinflammation (For review, see Hein and O'Banion, 2009), this reduction could be related to the improvements in the object recognition task and the Morris water maze task, representing declarative memory in mice (Figures 4, 5). We observed an apparently greater number of GFAP-positive cells regardless of age and cilostazol administration, in hippocampus compared to cerebral cortex (Zhang et al., 2017). Proliferation of astrocytes is a key indicator of oxidative stress (Moghaddam et al., 2014). Because the hippocampus has been reported to be one of the brain regions highly susceptible to oxidation (Dubey et al., 1996; Wang and Michaelis, 2010), increased numbers of GFAP-positive cells in hippocampus might be resulted from the modification by oxidative stress (Kaneko et al., 2002). The anti-inflammatory mechanism of cilostazol is yet to be fully elucidated. However, the increased cAMP levels associated with cilostazol administration may attenuate inflammatory responses through the cAMP-CREB signaling pathway (Aizawa et al., 2003). Among the genes regulated by CREB-dependent manner, brain-derived neurotrophic factor (BDNF), which is highly expressed in the hippocampus (Conner et al., 1997), is known as the modulator of inflammatory responses (Xu et al., 2017) and cognitive functions (for review, see Barros et al., 2019). Considering that cilostazol increased the BDNF-expressing glial cells in the hippocampus (Tanaka et al., 2010), it might be plausible that the anti-inflammatory effect of cilostazol might be manifested through the upregulation of BDNF. Another possibility is that the antiplatelet properties of cilostazol might be involved in the anti-inflammatory response, as coagulation produces excessive oxidants in capillary blood flow (Gregg et al., 2004). This, in turn, leads to elevated inflammatory responses (Mittal et al., 2014). Further research is required to uncover the mechanisms underlying the reduction of oxidative stress by cilostazol.

Microglial activation leads to increased [^18^F]FDG uptake in aged mice (Brendel et al., 2017). This finding and reduction of neuroinflammation by cilostazol administration (Figures 8E, F) led us to hypothesize that [^18^F]FDG uptake could be reduced by cilostazol. Cilostazol administration, however, increased the uptake of [^18^F]FDG throughout the brain (Figures 9A, B). The SUV calculation used in this study is a simple method to quantify cerebral uptake of [^18^F]FDG. However, it might not reflect exact rates of glucose metabolism in brain parenchyma because [^18^F]FDG uptake can be affected by several factors such as glucose transporters (Patching, 2015), cerebral blood flow (Bentourkia et al., 2000), and blood-brain barrier (BBB) states (Chiaravalloti et al., 2016). Evidence supporting this hypothesis includes the finding that the brain region with increased [^18^F]FDG uptake generally correspond to the region where the blood flow is abundant (Sakurada et al., 1978). In addition, the level of functional activity in the CNS regulates the rate of glucose metabolism, and blood flow is adjusted to the local metabolic demand (Sokoloff, 1981). Therefore, we encountered technical difficulties in dissociating the contribution of glucose metabolism and blood flow on functional activity in the CNS. PDE3s degrade both cAMP and cGMP efficiently (Degerman et al., 1987). PDE3s are mainly implicated in cardiovascular functions (Shakur et al., 2001) because of its distribution in platelets, heart, and vascular smooth muscle. Thus, the cilostazol-enhanced cGMP signaling potentially contributes to the increased [^18^F]FDG uptake through nitric oxide-cGMP signaling pathway to relax vascular smooth muscle (Schlossman and Desch, 2009). Closely related to these findings, cilostazol improves BBB integrity (Takagi et al., 2015; Edrissi et al., 2016; Yanai et al., 2017), keeps blood from coagulation (Ikeda, 1999), and increases cerebral blood flow (Bentourkia et al., 2000; Mochizuki et al., 2001). The positive correlation between cerebral blood flow and cognitive functions (Bracko et al., 2020) also supports the association of cognitive enhancement (Figure 4B, Figure 5C) and the increased cerebral blood flow. Therefore, it might be reasonable to consider that cilostazol exerts its favorable effect on the uptake of [^18^F]FDG through cGMP-mediated mechanisms and also cAMP-mediated ones. In an ongoing clinical trial, the effect of cilostazol is evaluating in patients of vascular cognitive impairment (NCT01872858). Because antiplatelet agent aspirin is used as comparator in the clinical trial, the diverse role of direct and indirect effects on cilostazol-induced reversal of cognitive function will be revealed. Further experiments that include more quantitative measurements of the cerebral glucose metabolic rate (CMRglc) (Moore et al., 2000) are required in order to clearly elucidate the mechanism that underlies the increased [^18^F]FDG brain uptake we observed following cilostazol administration.

Pre-dementia phases such as cognitive frailty is thought to be potentially reversible and preventable until probable point when neuronal damage becomes fatal (Kelaiditi et al., 2013; Ruan et al., 2015), however, symptomatic therapy is usually initiated upon the appearance of impaired cognitive function (Dey et al., 2017). As the aged population grows worldwide especially in developed countries (Prince et al., 2015; Gauthier et al., 2021), therapeutic intervention is an urgent goal to slow and/or delay the onset of cognitive impairment (de Magalhães et al., 2012; Endo, 2012; Yanai and Endo, 2015; Prickaerts et al., 2017). In addition to the beneficial effects of cilostazol to maintain memory functions (Yanai et al., 2018), our present results revealed that one-month-administration of cilostazol reverses the age-related memory impairment at a time when a deficit would normally be manifest. Cilostazol has been routinely prescribed around the world for the treatment of chronic peripheral arterial occlusion (O'Donnell et al., 2009) and intermittent claudication (Dawson et al., 1998; Chapman and Goa, 2003). Also, its safety profile is well established. In addition to these peripheral actions, cilostazol also reverses cognitive impairment in several rodent models, and it reduces cognitive impairment related to chronic cerebral hypoperfusion (Lee et al., 2006; Watanabe et al., 2006; Hiramatsu et al., 2010; Park et al., 2011; Godinho et al., 2015; Kitamura et al., 2017). In preliminary clinical studies using human patients, cilostazol is also demonstrated to be effective for treating mild cognitive impairment (Taguchi et al., 2013; Ihara et al., 2014). Cilostazol might approve to be a novel therapeutic intervention aimed at preventing or delaying cognitive impairment that is so prevalent in an ever-increasing aging population. In the clinical trials tested the efficacy of cilostazol in Alzheimer’s disease patients with white matter lesions, cilostazol administration preserved the regional glucose metabolism that is correlated with the improvement of the Alzheimer’s disease assessment scale-cognitive score (NCT01409564). However, other cognitive tasks including mini-mental state exam, Alzheimer’s disease cooperative study-activities of daily living inventory, and the clinical dementia rating sum of boxes, did not differ from the placebo group. Selective improvement in cognitive tasks by cilostazol administration was also reported in patients with Alzheimer’s disease (Sakurai et al., 2013). Considering the results obtained in the present study and in the clinical studies, beneficial effect of cilostazol on cognition may depend on the severity of the impairment.

In the present study, male mice were used based on our previous data on the detailed characterization of drug-free age-related functional changes in behavioral phenotype (Yanai and Endo, 2021) and on the effect of cilostazol administration (Yanai et al., 2014; Yanai et al., 2018). Male mice have long been used in accordance with a tradition in scientific research (Beery and Zucker, 2011), however, such a male bias has to be changed. As evident in human studies, prevalence of dementia is higher in females than in males (Zhu et al., 2021), and therapeutic efficacy of cholinesterase inhibitor is stronger and more beneficial for males (Haywood and Mukaetova-Ladinska, 2006; Davis and Barrett, 2009). In mouse model of aging, emerging evidence reveal the substantial sex-based differences in mice behavior (Jonasson, 2005; Spröwitz et al., 2013; Matsuda et al., 2015; Tucker et al., 2016). Furthermore, specific interactions between cholinesterase inhibitors and sex hormones (Wang et al., 2000; Smith et al., 2015) might affect therapeutic efficacy of the drugs between the sexes (Alves-Amaral et al., 2010). As balanced population of male and female mice are advocated in scientific research (Wald and Wu, 2010; Shansky, 2019), elucidating sex differences in behavioral phenotype and in therapeutic efficacy will be useful for the development of sex-sensitive strategies in the prevention and treatment of age-related memory impairment.

## Data Availability

The original contributions presented in the study are included in the article/Supplementary Material, further inquiries can be directed to the corresponding author.

## Abbreviations

BDNF, brain-derived neurotrophic factor; cAMP, 3’, 5’-cyclic adenosine monophosphate; cGMP, 3′, 5′-cyclic guanosine monophosphate; CNS, central nervous system; CREB, cAMP response element-binding protein; DG, dentate gyrus; [^18^F]FDG, 2-deoxy-2-[^18^F]fluoro-D-glucose; GFAP, glial fibrillary acidic protein; Iba1, ionized calcium-binding adapter molecule 1; IL, interleukin; NF-κB, nuclear factor kappa B; PDE, phosphodiesterase; PET, positron emission tomography; PKA, protein kinase A; SUV, standardized uptake value.
