# Supplementary material for: Reversal of spatial memory impairment by phosphodiesterase 3 inhibitor cilostazol is associated with reduced neuroinflammation and increased cerebral glucose uptake in aged male mice
Source: Front Pharmacol. 2022 Dec 21;13:1031637. doi: 10.3389/fphar.2022.1031637 (PMC9810637; doi:10.3389/fphar.2022.1031637)
Supplement: Supplementary file 1 [file DataSheet1.docx]

Supplementary Material

# The influence of cilostazol on [^18^F]FDG uptake in the brain

Aged 22-month-old mice were administered cilostazol (0% or 1.5% cilostazol in the feed) for one month, before undergoing 2-deoxy-2-[^18^F]-fluoro-D-glucose ([^18^F]FDG)- positron emission tomography (PET). The purpose was to assess the uptake of [^18^F]FDG. Mice fasted overnight and then were injected intraperitoneally with 35.8±1.8 MBq of [^18^F]FDG. Forty minutes after the injection, PET data acquisition of the brain was conducted for 12 min using a semiconductor small animal PET scanner (MIP-100; Sumitomo Heavy Industries). The mice were anesthetized with 1.5-2.0% isoflurane anesthesia during the entire scanning period. [^18^F]FDG uptake in each region was corrected for the injected dose and each mouse’s body weight and quantified as standardized uptake value (SUV; activity measured per mL of tissue/injected activity/g body weight).

**Figure S1. [^18^F]FDG uptake in the brain of cilostazol-administered aged mice.**

After the mixed-in-feed administration of cilostazol (control [0%] or 1.5%) for one month, 23-month-old mice were examined for the uptake of [^18^F]FDG. Forty minutes after [^18^F]FDG injection, PET data acquisition of the brain was conducted for 12 min. Scatter dot plot representing the uptake level of [^18^F]FDG (SUV) in the following brain: striatum (STR), cerebral cortex (CRX), thalamus (THA), cerebellum (CB), basal forebrain and septum (BFS), hypothalamus (HYP), amygdala (AMY), brain stem (BS), cingulated cortex (CG), superior colliculi (SC), olfactory bulb (OLF), mid brain (MID), and inferior colliculi (IC). Cilostazol administration significantly increased [^18^F]FDG uptake in almost all brain regions examined (Student’s t-test). The concentration of cilostazol in the feed was 0% and 1.5%. The control (0%) mice served as a non-drug control. **p* < 0.05, †*p* < 0.10. SUV, standardized uptake value (activity measured per mL of tissue/injected activity/g body weight). Horizontal bars indicate mean values.

# Table S1. Summary of statistical analysis

|  |  |
| --- | --- |
| Analysis / Task | Statistical value |
| Pharmacokinetic assay *** |  |
| main effect of dose | F (1, 20) = 8.76, p < 0.01 |
| main effect of time | F (2, 20) = 0.95, p = 0.403 |
| interaction (dose x time) | F (2, 20) = 0.89, p = 0.425 |
|  |  |
|  |  |
| Home-cage activity ** |  |
| Distance traveled |  |
| main effect of dose | F (2, 17) = 0.39, p = 0.683 |
| main effect of light condition | F (1, 17) = 89.10, p < 0.001 |
| interaction (dose x light condition) | F (2, 17) = 1.98, p = 0.169 |
|  |  |
| Food intake |  |
| main effect of dose | F (2, 24) = 1.47, p = 0.249 |
| main effect of light condition | F (1, 24) = 103.64, p < 0.001 |
| interaction (dose x light condition) | F (2, 24) = 0.65, p = 0.531 |
|  |  |
| Water intake |  |
| main effect of dose | F (2, 24) = 0.17, p = 0.846 |
| main effect of light condition | F (1, 24) = 178.22, p < 0.001 |
| interaction (dose x light condition) | F (2, 24) = 0.04, p = 0.958 |
|  |  |
|  |  |
| Open field test ** |  |
| Center time |  |
| main effect of dose | F (2, 43) = 0.96, p = 0.392 |
| main effect of light condition | F (1, 43) = 11.79, p < 0.001 |
| interaction (dose x light condition) | F (2, 43) = 0.34, p = 0.711 |
|  |  |
| Immobile time |  |
| main effect of dose | F (2, 43) = 1.29, p = 0.287 |
| main effect of light condition | F (1, 43) = 23.83, p < 0.001 |
| interaction (dose x light condition) | F (2, 43) = 2.61, p = 0.085 |
|  |  |
| Distance traveled |  |
| main effect of dose | F (2, 43) = 1.73, p = 0.190 |
| main effect of light condition | F (1, 43) = 7.60, p < 0.01 |
| interaction (dose x light condition) | F (2, 43) = 1.88, p = 0.164 |
|  |  |
| Number of rearings |  |
| main effect of dose | F (2, 43) = 3.39, p < 0.05 |
| main effect of light condition | F (1, 43) = 30.73, p < 0.001 |
| interaction (dose x light condition) | F (2, 43) = 2.09, p = 0.136 |
|  |  |
|  |  |
| Object recognition test ** |  |
| Spatial change test |  |
| main effect of dose | F (2, 43) = 5.18, p < 0.01 |
| main effect of object category | F (1, 43) = 2.44, p = 0.125 |
| interaction (dose x object category) | F (2, 43) = 5.48, p < 0.01 |
|  |  |
| Object change test |  |
| main effect of dose | F (2, 43) = 0.96, p = 0.392 |
| main effect of object category | F (1, 43) = 31.73, p < 0.001 |
| interaction (dose x object category) | F (2, 43) = 0.27, p = 0.766 |
|  |  |
|  |  |
| Morris water maze task |  |
| Swim speed * |  |
| Spatial acquisition test | F (2, 41) = 1.10, p = 0.341 |
| Probe test | F (2, 41) = 0.21, p = 0.815 |
| Cued training | F (2, 41) = 1.95, p = 0.156 |
|  |  |
| Spatial acquisition test (latency)** |  |
| main effect of dose | F (2, 41) = 1.09, p = 0.345 |
| main effect of training day | F (11, 451) = 22.94, p < 0.001 |
| interaction (dose x training day) | F (22, 451) = 1.49, p = 0.071 |
|  |  |
| Spatial acquisition test (distance)** |  |
| main effect of dose | F (2, 41) = 0.12, p = 0.891 |
| main effect of training day | F (11, 451) = 18.15, p < 0.001 |
| interaction (dose x training day) | F (22, 451) = 1.48, p = 0.076 |
|  |  |
| Probe test-platform crossing * |  |
| main effect of dose | F (2, 41) = 4.40, p < 0.05 |
|  |  |
| Probe test-quadrant time * |  |
| main effect of dose | F (2, 41) = 0.05, p = 0.956 |
|  |  |
| Cued training (latency)** |  |
| main effect of dose | F (2, 41) = 5.26, p < 0.01 |
| main effect of training day | F (3, 123) = 40.88, p < 0.001 |
| interaction (dose x training day) | F (6, 123) = 1.46, p = 0.197 |
|  |  |
| Cued training (distance)** |  |
| main effect of dose | F (2, 41) = 2.41, p = 0.103 |
| main effect of training day | F (3, 123) = 10.15, p < 0.001 |
| interaction (dose x training day) | F (6, 123) = 2.09, p = 0.059 |
|  |  |
|  |  |
| Pavlovian fear conditioning task * |  |
| Cue-dependent memory test (1 hr) | F (2, 40) = 2.04, p = 0.143 |
| Cue-dependent memory test (24 hrs) | F (2, 40) = 0.91, p = 0.412 |
| Context-dependent memory test (48 hrs) | F (2, 40) = 0.31, p = 0.736 |
|  |  |
|  |  |
| Hotplate test * | |
| main effect of dose | F (2, 19) = 0.37. p = 0.696 |
|  |  |
|  |  |
| Electrical footshock sensitivity test * |  |
| Pawflick | F (2, 19) = 0.47. p = 0.631 |
| Vocalization | F (2, 19) = 0.60. p = 0.560 |
|  |  |
|  |  |
| Immunohistochemical staining * |  |
| Iba1 |  |
| CA1 | F (2, 17) = 9.21, p < .01 |
| CA3 | F (2, 17) = 11.95, p < .001 |
| DG | F (2, 17) = 9.96, p < .001 |
| Cerebral cortex | F (2, 17) = 19.19, p < .001 |
|  |  |
| GFAP |  |
| CA1 | F (2, 17) = 7.67, p < .01 |
| CA3 | F (2, 17) = 10.46, p < .001 |
| DG | F (2, 17) = 34.28, p < .001 |
| Cerebral cortex | F (2, 17) = 4.43, p < .05 |
|  |  |
|  |  |
| [^18^F]FDG # |  |
| Hippocampus | t (15) = 2.59, p < 0.05 |
| Whole brain | t (15) = 2.46, p < 0.05 |
| Striatum | t (15) = 2.81, p < 0.05 |
| Cortex | t (15) = 2.66, p < 0.05 |
| Thalamus | t (15) = 2.65, p < 0.05 |
| Cerebellum | t (15) = 2.86, p < 0.05 |
| Basal forebrain and septum | t (15) = 2.19, p < 0.05 |
| Hypothalamus | t (15) = 1.20, p = 0.249 |
| Amygdala | t (15) = 2.11, p = 0.052 |
| Brain stem | t (15) = 1.10, p = 0.290 |
| Cingulate cortex | t (15) = 2.84, p < 0.05 |
| Superior colliculi | t (15) = 2.69, p < 0.05 |
| Olfactory bulb | t (15) = 2.43, p < 0.05 |
| Midbrain | t (15) = 1.97, p = 0.068 |
| Inferior colliculi | t (15) = 2.59, p < 0.05 |
| Blood glucose level | t (15) = 0.86, p = 0.403 |

* One-way ANOVA

** Two-way repeated measures ANOVA

*** Two-way factorial ANOVA

# t-test

DG, dentate gyrus; [^18^F]FDG, 2-deoxy-2-[^18^F]fluoro-D-glucose; GFAP, glial fibrillary acidic protein; Iba1, ionized calcium-binding adapter molecule 1; PET, positron emission tomography.
